# Functional convergence of histone methyltransferases EHMT1 and KMT2C involved in intellectual disability and autism spectrum disorder

**DOI:** 10.1371/journal.pgen.1006864

**Published:** 2017-10-25

**Authors:** Tom S. Koemans, Tjitske Kleefstra, Melissa C. Chubak, Max H. Stone, Margot R. F. Reijnders, Sonja de Munnik, Marjolein H. Willemsen, Michaela Fenckova, Connie T. R. M. Stumpel, Levinus A. Bok, Margarita Sifuentes Saenz, Kyna A. Byerly, Linda B. Baughn, Alexander P. A. Stegmann, Rolph Pfundt, Huiqing Zhou, Hans van Bokhoven, Annette Schenck, Jamie M. Kramer

**Affiliations:** 1 Department of Human Genetics, Radboudumc, Nijmegen, The Netherlands; 2 Radboud Institute of Molecular Life Sciences, Nijmegen, The Netherlands; 3 Donders Institute for Brain, Cognition, and Behaviour, Centre for Neuroscience, Nijmegen, The Netherlands; 4 Department of Biology, Faculty of Science, Western University, London, Ontario, Canada; 5 Division of Genetics and Development, Children’s Health Research Institute, London, Ontario, Canada; 6 Department of Clinical Genetics and School for Oncology & Developmental Biology (GROW), Maastricht University Medical Center, Maastricht, the Netherlands; 7 Department of Pediatrics, Máxima Medical Centre, Veldhoven, The Netherlands; 8 Clinical Genetics and Metabolism, Children's Hospital Colorado, Aurora, Colorado; 9 Department of Laboratory Medicine and Pathology, Mayo Clinic, Rochester, Minnesota; 10 Department of Molecular Developmental Biology, Radboud University, Nijmegen, The Netherlands; 11 Department of Physiology and Pharmacology, Schulich School of Medicine and Dentistry, Western University, London, Ontario, Canada; MRC Human Genetics Unit, UNITED KINGDOM

## Abstract

Kleefstra syndrome, caused by haploinsufficiency of *euchromatin histone methyltransferase 1* (*EHMT1*), is characterized by intellectual disability (ID), autism spectrum disorder (ASD), characteristic facial dysmorphisms, and other variable clinical features. In addition to *EHMT1* mutations, *de novo* variants were reported in four additional genes (*MBD5*, *SMARCB1*, *NR1I3*, and *KMT2C*), in single individuals with clinical characteristics overlapping Kleefstra syndrome. Here, we present a novel cohort of five patients with *de novo* loss of function mutations affecting the histone methyltransferase KMT2C. Our clinical data delineates the *KMT2C* phenotypic spectrum and reinforces the phenotypic overlap with Kleefstra syndrome and other related ID disorders. To elucidate the common molecular basis of the neuropathology associated with mutations in *KMT2C* and *EHMT1*, we characterized the role of the *Drosophila* KMT2C ortholog, trithorax related (trr), in the nervous system. Similar to the *Drosophila* EHMT1 ortholog, G9a, trr is required in the mushroom body for short term memory. Trr ChIP-seq identified 3371 binding sites, mainly in the promoter of genes involved in neuronal processes. Transcriptional profiling of pan-neuronal *trr* knockdown and *G9a* null mutant fly heads identified 613 and 1123 misregulated genes, respectively. These gene sets show a significant overlap and are associated with nearly identical gene ontology enrichments. The majority of the observed biological convergence is derived from predicted indirect target genes. However, trr and G9a also have common direct targets, including the *Drosophila* ortholog of Arc (Arc1), a key regulator of synaptic plasticity. Our data highlight the clinical and molecular convergence between the KMT2 and EHMT protein families, which may contribute to a molecular network underlying a larger group of ID/ASD-related disorders.

## Author summary

Neurodevelopmental disorders (NDDs) like intellectual disability (ID) and autism spectrum disorder (ASD) present an enormous challenge to affected individuals, their families, and society. Understanding the mechanisms underlying NDDs may lead to the development of targeted therapeutics, but this is complicated by the great clinical and genetic heterogeneity seen in patients. Mutations in hundreds of genes have been implicated in NDDs, giving rise to diverse clinical presentations. However, evidence suggests that many of these genes lie in common biological pathways, and mutations in genes that are involved in similar biological functions give rise to more similar clinical phenotypes. Here, we define a novel ID disorder with comorbid ASD (ID/ASD) caused by mutations in *KMT2C*. This disorder is defined by clinical features that overlap with a group of other disorders, including Kleefstra syndrome, which is caused by *EHMT1* mutations. In the fruit fly, we show that the KMT2 and EHMT protein families regulate a highly converging set of biological processes. Both *EHMT1* and *KMT2C* encode histone methyltransferases, which regulate gene transcription by modifying chromatin structure. Further understanding of the common gene regulatory networks associated with this group of ID- and ASD-related disorders may lead to the identification of novel therapeutic targets.

## Introduction

Kleefstra syndrome (OMIM #610253) is a neurodevelopmental disorder that is caused by haploinsufficiency of *EHMT1* [1, 2]. *EHMT1* encodes a histone methyltransferase that regulates gene expression through modification of chromatin structure and through interactions with other transcription factors [2–4]. The core phenotype of Kleefstra syndrome is characterized by intellectual disability (ID), childhood hypotonia, and distinctive facial characteristics. Autism spectrum disorder (ASD) and other behavioural problems such as sleep disturbances and feeding difficulties are also frequently observed [5, 6]. Kleefstra syndrome shares considerable phenotypic overlap with several other disorders characterized by ID, ASD, behavioural problems, and hypotonia [7, 8]. These disorders include Pitt-Hopkins syndrome, Smith-Magenis syndrome, Rett syndrome, *MBD5* deletion/duplication, and Angelman syndrome. We have collected a cohort of individuals with clinical characteristics that fit within this spectrum. In the pre-exome sequencing era, around 25% of these cases were positive for *EHMT1* haploinsufficiency characterizing Kleefstra syndrome [9]. Previously, we hypothesized that these unsolved, *EHMT1* mutation-negative individuals with overlapping clinical features may carry mutations in other genes that code for proteins acting in EHMT1-related molecular pathways or biological processes.

Genetic analyses of four individuals within our cohort using next generation sequencing methods revealed potentially causative *de novo* mutations in four genes (*MBD5*, *SMARCB1*, *NR1I3*, and *KMT2C*), all of which encode proteins involved in regulation of gene expression and/or chromatin structure [9]. Available physical interaction data between these proteins [10–14] and pairwise genetic interactions identified between the *Drosophila* orthologs of these genes [9], supported the hypothesis that *EHMT1* and the genes identified in the individuals presenting with clinical overlap act in shared molecular pathways. In particular, a very strong antagonistic genetic interaction was observed between the *Drosophila EMHT1* ortholog, *G9a*, and the *KMT2C* ortholog, *trithorax related* (*trr*), in the context of *Drosophila* wing development [9]. Genetic combination of *G9a* overexpression together with *trr* knockdown led to a complete developmental arrest and death of the targeted wing tissue, while the misregulation of either gene alone had only subtle effects on wing morphology [9]. Although these data argued for a conserved functional relationship between the two genes, the underlying shared molecular basis remained unknown.

G9a and trr are both histone methyltransferases, but they have different substrates. G9a mediates mono- and dimethylation of histone H3 at lysine 9 (H3K9me2). This is generally regarded as a repressive histone mark, however, in mammals the EHMT1 protein has also been shown to activate gene expression independent of its methyltransferase activity [15, 16]. *G9a* mutant flies are fully viable [17, 18] but do show phenotypic differences compared to wildtype flies. Loss of *Drosophila G9a* delays embryonic development [19]. In larval stages, *G9a* mutants show defects in the morphology of multidendrite neurons and have altered crawling behaviour [18]. Adult *G9a* mutants have defects in habituation learning, short and long term courtship memory, and show decreased tolerance to virus infections [18, 20]. Genome-wide identification of genomic regions with reduced H3K9me2 in *G9a* mutant larvae revealed a large number of target sites in genes that are strongly enriched for neuronal functions [18]. In mouse knockout models, loss of *Ehmt1* causes phenotypes that are reminiscent of Kleefstra Syndrome, including deficits in learning and memory, increased anxiety, hypotonia, cranial abnormalities, and developmental delay [21–24]. These studies show that the EHMT family of proteins are evolutionarily conserved regulators of neurodevelopmental processes and cognition. However, the underlying molecular mechanisms and functional partners of EHMT proteins in these processes are poorly understood.

Studies investigating the molecular biology of *Drosophila* trr and its binding partners have revealed a potential dual function for this protein in the regulation of gene expression. Trr mediates mono- and tri-methylation of histone H3 at Lysine 4 (H3K4me1 and H3K4me3) [14, 25], histone modifications that are found at enhancers and active gene promoters, respectively [26]. Trr and its mammalian orthologs KMT2C and KMT2D, are present in a conserved COMPASS-like (complex of proteins associated with Set1) protein complex that mediates H3K4me1 at enhancers [27, 28]. Additionally, trr interacts with the ecdysone receptor and is a co-activator of ecdysone-mediated transcription that deposits H3K4 trimethylation at promoters [14]. The role of trr in the nervous system and how it relates to G9a is unknown.

Here, we describe the first human cohort with *de novo* loss of function mutations in the *trr* ortholog, *KMT2C*, allowing us to delineate the phenotypic spectrum and define the core phenotype associated with mutations in this gene. In agreement with the single previously reported patient, these individuals show clinical overlap with Kleefstra syndrome and other related neurodevelopmental disorders; reemphasizing (1) an important role for *KMT2C* in neurodevelopment, and (2) a biological link with EHMT1. We further show that *Drosophila* trr shares the essential role in neurodevelopment with its human ortholog, and provide evidence for biological convergence between trr and G9a.

## Results

### *De novo* mutations in *KMT2C* cause ID and ASD

**Fig 1:**
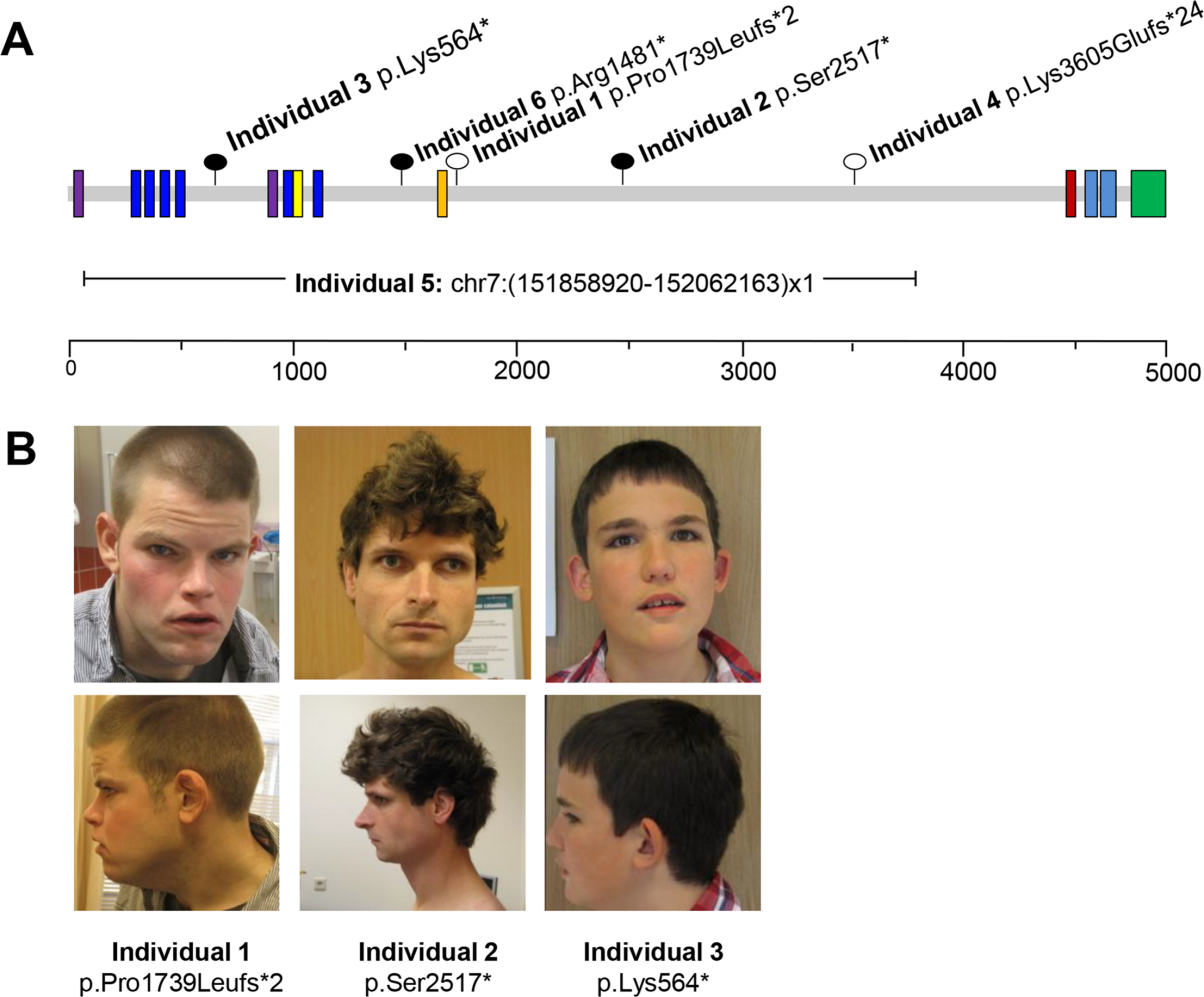
Patients and identified *KMT2C* mutations. (A) Schematic view of the KMT2C protein with reported domains (purple: AT hook DNA binding domain; dark blue: zinc finger domain; yellow: cysteine rich; orange: High mobility group (HMG); dark red: Ring finger; light blue: "FY-rich" domain; dark green: SET and Post-SET domains) and identified frameshift mutations (open lollipops), nonsense mutations (closed lollipops) and deletion. Scale bar represents amino acid position. (B) Frontal and lateral photographs of individual 1 at age 29 years, individual 2 at age 31 years, and individual 3 at age 15 years. Though there are variable facial features, dysmorphisms consistent with Kleeftra syndrome are observed, including flattened midface (individual 1 and 4), prominent eyebrows (individuals 1 and 3), everted lower lip (individual 4), and thick ear helices (individuals 1 and 3). Photographs of individual 4 and 5 are not shown.

Through the large scale application of diagnostic whole exome sequencing for unexplained neurodevelopmental disorders in the clinical genetics centers at the Radboudumc and Maastricht UMC [29], we identified five *de novo KMT2C* mutations. All these mutations are predicted to cause loss of function, including c.5216del (p.Pro1739Leufs*2) in individual 1, c.7550C>G (p.Ser2517*) in mosaic (≈30% of blood cells) in individual 2, c.1690A>T (p.Lys564*) in individual 3, and c.10812_10815del (p.Lys3605fs) in individual 4 (**Fig 1A**). Using microarray-based comparative genomic hybridization for patient 5, an intragenic 203kb *de novo* deletion (Chr7: 151858920–152062163)x1 was identified (**Fig 1A**) and confirmed by locus-specific qPCR.

Identification of these five novel mutations, in addition to the previously published case [9], has allowed us to establish the clinical phenotype associated with *KMT2C* mutations (**Table 1**). All individuals had ID, ranging from mild to severe, language and motor delay, and autism or Pervasive Developmental Disorder (PDD), a condition in the autistic spectrum. Other recurrent clinical features were short stature (2/6), microcephaly (3/6), childhood hypotonia (3/6), kyphosis/scoliosis (3/6) and recurrent respiratory infections (2/6). Kleefstra-like facial dysmorphisms, including flattened midface, prominent eyebrows, everted lower lip, and thick ear helices, were observed in several individuals (**Fig 1B**).

**Table 1:** Summary of molecular and clinical features of individuals with *KMT2C* mutations.

	Individual 1	Individual 2	Individual 3	Individual 4	Individual 5	Individual 6 Kleefstra et al.(2012)
Gender	Male	Male	Male	Female	Female	Female
Age of examination	29 years	31 years	15 years	7 years	10 years	15 years
**Mutation (NM_170606.2)**						
Chromosome position (Hg19)	g.151880108del	g.151874988G>C	g.151947983T>A	g.151859847_151859850del	-	g.151891591G>A
cDNA change	c.5216del	c.7550C>G	c.1690A>T	c.10812_10815del	-	c.4441C>T
Amino acid change	p.(Pro1739Leufs*2)	p.(Ser2517*)	p.(Lys564*)	p.(Lys3605Glufs*24)	-	p.(Arg1481*)
Mosaic	-	+ (30% blood)	-	-	-	-
Deletion	-	-	-	-	7q36.1 (151858920–152062163)x1	-
Additional *de novo* mutations	*PHF21A*^1^	*UBR5*^2^ *C11orf35*^3^	-	-	-	-
**Growth**						
Height	171.5 cm (-1.7 SD)	179 cm (-0.5 SD)	160 cm (-2 SD)	109 cm (-3 SD)	N/A	148 cm (-2.5 SD)
Weight	63.5 kg (+0.6 SD)	53.8 kg (-1.5 SD)	55 kg (+1,7SD)	16 kg (-1.5 SD)	20 kg (-2.5 SD)	41 kg (0 SD)
Head circumference	56.6 cm (-0.5 SD)	57 cm (-0.5 SD)	55 cm (-0,6 SD)	47.5 cm (-2.25 SD)	49.5 cm (-2 SD)	52 cm (-2 SD)
**Development**						
Intellectual disability	+ Moderate	+ Mild	+ Moderate–IQ 50	+ Mild—IQ63	+ Severe	+ Moderate—IQ 35
Language and motor delay	+	+	+	+	+	+
**Neurological**						
Behavior problems	+ Autistic-traits	+ Autism	+ PDD-NOS, ADHD	+ Autism, sleeping disorder	+ Automutilation	+ hyperactivity, aggressiveness
Childhood hypotonia	-	-	+	-	+	+
Epilepsy	+	-	-	-	+	-
**Skeletal**						
Kyphosis/Scoliosis	+ (thoracal kyphosis)	+ (Scoliosis)	-	-	+ (Kyphosis)	-
**Other**						
	PKU, RRI	Strabismus, cryptorchidism	Bifid uvula, hypospadia, bilateral inguinal hernia	RRI, dry skin, hoarse voice	Plagiocephaly	-
**MRI**						
	Not performed	N/A	Normal	Normal	Non-progressive enlarged extracerebral space	N/A

Abbreviations: N/A = not available; PKU = phenylketonuria; RRI = recurrent respiratory infections

^1^ *PHF21A*: c.1956del; p.(Ala653Profs*103)

^2^ *UBR5*: c.5720G>A; p.(Arg1907His)

^3^ C11orf35; p.(Pro602Leu)

### The *Drosophila* KMT2C ortholog, trr, is required for short term courtship memory

**Fig 2:**
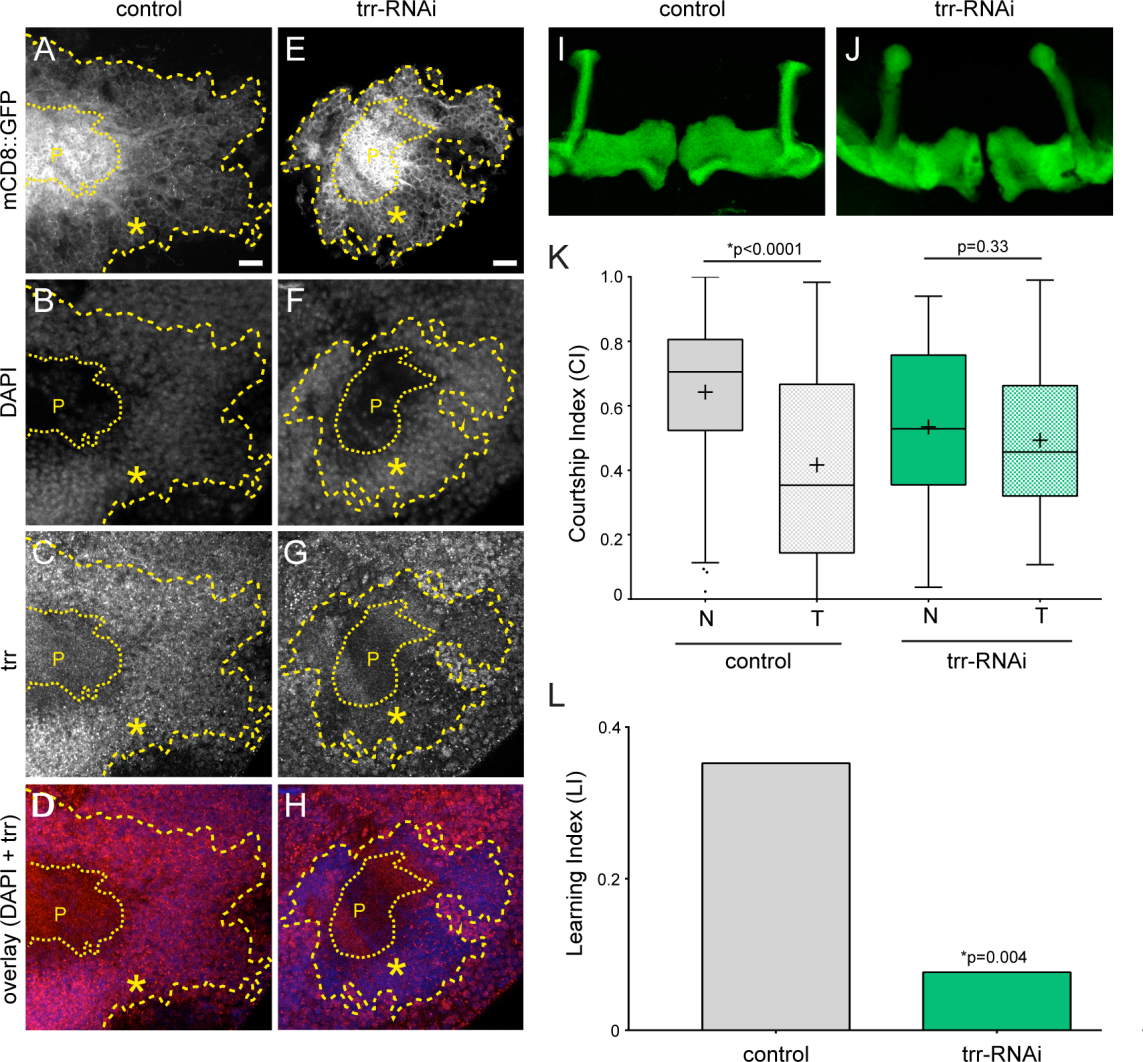
Trr is required in the mushroom body for short term memory. Fluorescent confocal images of control (A-D) and trr knockdown (E-H) adult male brains. UAS-mCD8::GFP calyx (A, E) shows the expression domain of the *R14H06-Gal4* driver in the mushroom body and is marked by yellow dashed lines and an asterisk. The scale bar represents 10 μm. DAPI (B, F) is shown to identify nuclei and note the low nuclear density in the peduncle, which is indicated with a P. Trr (C, G) is labeled by immunohistochemistry using an anti-trr antibody. The overlay of DAPI and trr signal (D, H) shows a reduction of trr in the target cells (blue and red channels). (I-J) Confocal projections showing the main axonal lobes of the mushroom body that are labeled by *UAS-mDC8*::*GFP* through expression with the *R14H06-Gal4* driver in control flies (I) and trr knockdown flies (J). The scale bar represents 10 μm. (K) Standard boxplots representing the courtship indexes (CIs) resulting from courtship conditioning in control and trr knockdown flies. + indicates the mean. The mean CI for naïve and trained flies was compared using the Mann-Whitney test. (L) Learning Indexes (LI) for controls and trr knockdown flies derived from the CIs. Trr knockdown males have a significantly reduced LI (randomization test, 10,000 bootstrap replicates).

To assess the functional role of KMT2C in neurons, we investigated the closest *Drosophila melanogaster* ortholog, trithorax related (trr), which shares a one-to-two evolutionary relationship with the human paralogs KMT2C and KMT2D [28]. Since homozygous mutations in *trr* are lethal [30], we used the UAS/Gal4 system [31] and inducible RNA interference (RNAi) [32] to assess the role of trr in the adult fly nervous system. Knockdown of *trr* was targeted specifically to the mushroom body (MB), the learning and memory center of the fly brain, using the *R14H06-Gal4* driver line from the Janelia FlyLight collection [33]. To estimate the knockdown efficiency under these conditions we co-expressed *UAS-trr-RNAi* with *UAS-mCD8*::*GFP* and performed immunohistochemistry using a trr antibody [34] (**Fig 2A–2H**). Trr protein is localized in the nuclei of the mushroom body calyx, as seen by colocalization with DAPI. A clear reduction of trr staining in cells expressing *trr-RNAi*, which are marked by *UAS-mCD8::GFP*, is observed (**Fig 2E–2H**). Under these conditions we observed no gross morphological defects in the MB upon *trr* knockdown (**Fig 2I and 2J**). Next, to test these MB-specific knockdown flies for defects in learning and memory we used a classic behaviour paradigm known as courtship conditioning. In this assay male flies exhibit a learned reduction of courtship behaviour after rejection by a non-receptive premated female [18, 35]. We tested short term memory by measuring the courtship index (CI) in naïve males compared to males exposed to sexual rejection for one hour by a premated female. Control flies expressed a transgenic RNAi construct targeting *mCherry*, which is inserted into the same genetic background as the *trr-RNAi*. These controls showed significant reduction in CI in rejected flies compared to naïve (**Fig 2K**). Flies expressing the *trr-RNAi* construct did not exhibit a significant reduction in CI in response to rejection (**Fig 2K**), and as a result, had a significantly lower learning index (LI) than the controls (**Fig 2L**). These data show that trr is required in the mushroom body for short term memory.

### Trr binds to the promoter of genes important for the nervous system

**Fig 3:**
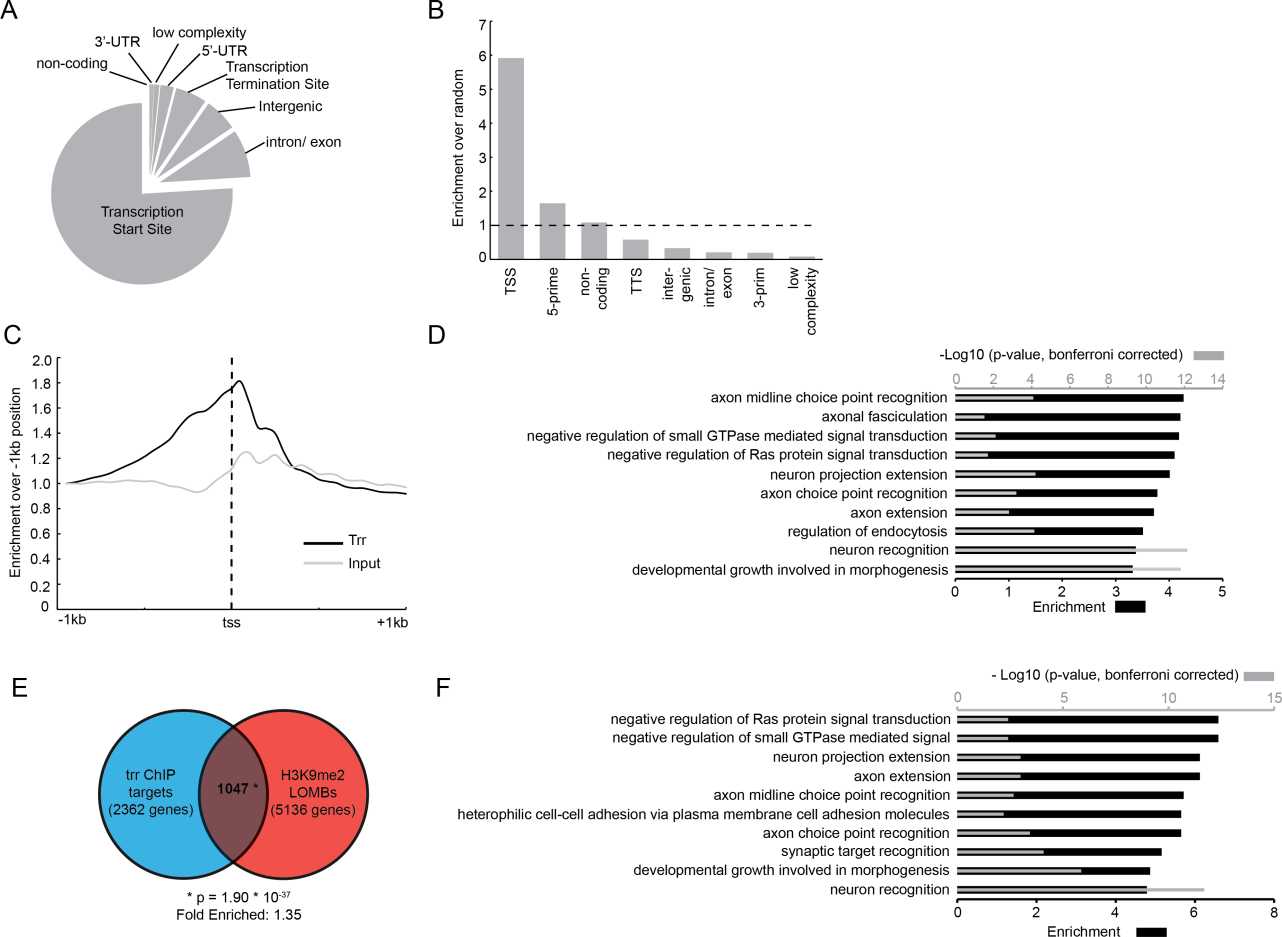
Trr localizes to promoters of neuronal genes in *Drosophila* heads and shows a significant overlap with G9a targets. (A) Pie-chart representing the location of trr binding sites in annotated genomic features as specified by HOMER software. (B) Fold enrichment of trr binding sites in annotated genomic features compared to an equivalent group of random genomic positions. (C) Average trr occupancy (black) of all transcription start sites (tss) relative to the –1kb region compared to the average read depth in the input control (grey). (D) Gene ontology enrichment analysis of genes with a trr binding site near the tss. Shown here are the top 10 enriched terms. (E) Venn diagram showing the overlap between predicted trr target genes identified here, and predicted G9a targets that were previously published [18]. The overlap of 1047 genes is larger than expected by random chance, based on a hypergeometric test (p-value = 1.9*10^−37^ and 1.35 times enriched). (F) Top 10 enriched GO terms for biological processes identified for the 1047 overlapping predicted targets for G9a and trr. For D and F, enrichment is indicated by black bars (lower x-axis), and the –log10 transformation of p-values is indicated by grey bars (upper x-axis).

Given the known role of trr as a transcriptional co-activator [14], we sought to identify genomic binding regions of trr to gain insight into its target genes and the processes that it regulates in the nervous system. To do this, we conducted chromatin immunoprecipitation combined with next generation sequencing (ChIP-seq) using wildtype adult *Drosophila* heads and a validated ChIP grade antibody directed against trr [14, 34]. After exclusion of poorly aligned and ambiguously mapped reads (**S1 Table**), the MACS2 (model based analysis of chip-seq) algorithm [36] was used to identify genomic regions that were significantly enriched for trr binding in two biological replicates (**S1 Table**). This analysis identified 3371 trr binding sites (**S2 Table**) covering 1.27% of the genome. About 75% of trr binding sites (2564) were located within 1kb up- or downstream of the transcription start sites (tss) of 2362 unique genes. The remaining 25% of trr binding sites were associated to other genomic features (low complexity, 5’- or 3’-prime region, transcription termination site, intron/exon), each at a low frequency (**Fig 3A, S2 Table**). Taking the genome-wide abundance of annotated features into account, trr binding at the tss is six fold enriched compared to random genomic positions (**Fig 3B**). The average trr occupancy profile over the tss of all genes shows a clear enrichment for trr when compared to the input control (**Fig 3C**). The observed association of trr with promoter regions in fly heads is consistent with previously published trr ChIP-seq data from cultured S2 cells [25]. Gene Ontology (GO) enrichment analysis of the 2362 unique genes with trr binding at the tss revealed a strong enrichment for neuronal terms, such as “axon extension” and “neuron recognition”. Also enriched was the term “negative regulation of Ras protein signal transduction”, a process that is corrupted in a series of ID disorders referred to as RASopathies [37] (**Fig 3D, S3 Table**). Taken together, our data demonstrate that, in fly heads, trr binds to the promoter of many genes involved in neuronal processes.

### G9a and trr show convergence in genomic targets

Given the clinical overlap between patients with *KMT2C* and *EHMT1* mutations (**Fig 1, Table 1**), and our previous studies showing a very strong genetic interaction between the two *Drosophila* orthologs [9], we reasoned that trr and G9a may regulate the expression of common genes and/or biological pathways. We investigated whether G9a and trr have common direct target genes (**Fig 3E and 3F)**. For this we compared the genes associated with trr binding sites identified here (**Fig 3A–3D**) with previously determined G9a target genes that were identified by investigating loss of H3K9me2 in G9a mutants [18]. This analysis revealed a significant overlap of 1047 genes based on hypergeometric probability (p < 1.9*10^−37^, fold change = 1.35, **Fig 3E**). These genes are primarily enriched for GO-terms associated with neuronal development and function, including “axon extension”, “neuron recognition”, and again, “negative regulation of Ras protein signaling” (**Fig 3F, S4 Table)**. This suggests that trr and G9a have the potential to directly regulate a common set of genes with important functions in neurons.

### G9a and trr regulate the expression of common genes and biological processes

**Fig 4:**
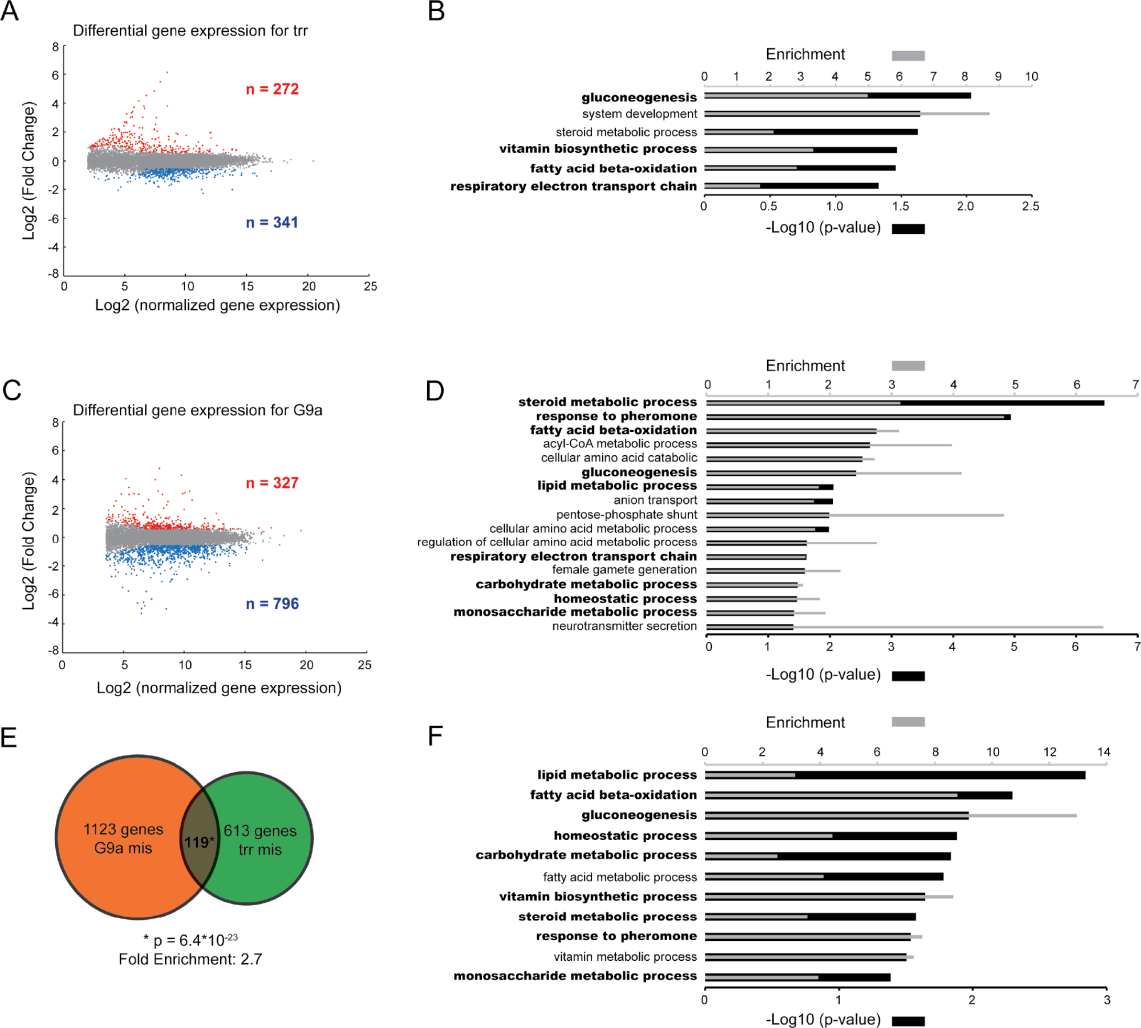
Differentially expressed genes in *trr* and *G9a* mutants show a strong biological overlap. (A,C) Scatter plots showing Log2 fold changes plotted against the Log2 normalized expression. Significantly up and down regulated genes (p-adj < 0.05, FC > 1.5) are represented by red and blue dots respectively in the G9a (A) and trr (C) mutant. Enriched GO terms are shown for differentially expressed genes in trr (B) and G9a (D) mutant heads. (E) Venn Diagram showing the overlap of differential expressed genes between the two mutant conditions. The overlap of 119 is significantly more than expected by random chance, based on a hypergeometric test (p-value = 6.4*10^−23^ and 2.7 times enriched). (F) GO term enrichment of the 119 overlapping genes. (B,D,F) Enriched GO terms were identified using the Panther software (GO-slim setting). The –log10 transformation of p-values is indicated by black bars (lower x-axis). Grey bars (upper x-axis) indicate enrichment. GO terms that overlap between the different datasets in panels B,D, and F, are indicated in bold.

To characterize the effect of *trr* and *G9a* mutations at the transcriptional level, we conducted whole-transcriptome mRNA sequencing (RNA-seq) on wildtype and mutant fly heads. Validation of pan-neuronal *trr* knockdown in fly heads was performed by RT-qPCR and revealed a 34% reduction in trr mRNA in whole fly heads, encompassing both the targeted neuronal knockdown cells and all other non-targeted cells (**S1 Fig**). Only reads aligned unambiguously to exons were considered for RNA-seq analysis (**S5 Table**). Upon trr knockdown, 613 genes were differentially expressed compared to controls (p-adj < 0.05, fold change > 1.5), with 341 genes downregulated, and 272 genes up regulated (**Fig 4A**, **S6 Table**). GO enrichment analysis of the differentially expressed genes in *trr* knockdown heads revealed mainly terms related to metabolism (**Fig 4B**). In *G9a* null mutant heads, 1123 genes were differentially expressed compared to controls (p-adj < 0.05, fold change > 1.5), with 796 genes downregulated and 327 genes upregulated (**Fig 4C, S7 Table**). These differentially expressed genes shared a striking overlap in GO enrichment when compared to differentially expressed genes in *trr* knockdown heads. In fact, 4 of the 6 GO terms that were enriched upon *trr* knockdown were also enriched in the analysis of *G9a* mutant heads (**overlapping GO terms in bold**, **Fig 4B and 4D**), including “gluconeogenesis”, “steroid metabolic processes”, “fatty acid beta oxidation”, and “respiratory electron transport chain”.

In addition to the overlapping enriched GO terms, we identified 119 differentially expressed genes represented in both mutant conditions (**Fig 4E and 4F**). This overlap is significantly larger than expected by random chance (p < 6.4*10^−23^, fold change = 2.7, hypergeometric test) (**Fig 4E**). GO enrichment analysis of these 119 genes again revealed an nearly identical set of enriched GO terms, with 9 of the 11 enriched terms represented in the analysis of all genes dysregulated in G9a and trr mutant conditions (**overlapping GO terms in bold**, **Fig 4F**). The striking overlap of specific GO terms associated with genes that are differentially expressed upon loss of trr and G9a suggest a high level of functional convergence between the two proteins.

Of the 119 commonly mis-regulated genes identified in *G9a* mutants and *trr* knockdown flies 5 genes were upregulated in both conditions, while 47 genes were downregulated in both conditions; 18 genes were upregulated in G9a and downregulated in trr, while 49 genes were downregulated in G9a and upregulated in trr (**S8 Table**). Although this distribution is not fully random, there is no clear pattern. The significant enrichment observed in the majority of the categories (**S8 Table**) suggests that many of the common differentially expressed genes may result from indirect regulation. Notably, only five of the overlapping genomic targets (**Fig 3E**, 1047 genes) are also found to be differentially expressed (**Fig 4E**, 119 genes). These five genes—*PCB*, *mTTF*, *Acer*, *Reg-2*, and *Arc1*—represent potential common direct targets of G9a and trr that could act as hub genes to account for the high degree of biological convergence among indirect targets (**S9 Table**, see discussion).

To further explore the origin of the biological convergence of differentially expressed genes in *trr* RNAi and *G9a* mutant heads, we classified these genes into “potentially direct” and “potentially indirect” targets. Potentially direct targets for trr, a transcriptional activator, were defined as genes with an associated trr binding site that were down regulated in *trr* RNAi flies. Of 341 downregulated genes, 47 fulfill these criteria. For G9a, direct targets were defined as genes with an associated loss of H3K9me2 (a repressive modification) in *G9a* mutants (as defined in Kramer *et al*. [18]) that were up regulated in *G9a* mutant heads. Of the 327 genes upregulated in *G9a* mutant heads, 84 were identified as potential direct G9a targets using these criteria. GO enrichment analysis of predicted direct targets revealed metabolic terms and several terms relevant to learning and memory, such as “sensory perception” and “cellular calcium ion homeostasis” (**S2 Fig**). However, no overlap in enriched GO terms was observed among the predicted direct targets of trr and G9a. In contrast, GO enrichment analysis of predicted indirect targets (**S2 Fig**) was nearly identical to that observed when all differentially expressed genes were analyzed (**Fig 4**) and the overlap of specific terms between G9a and trr datasets was even stronger (5 of the 6 GO terms that were enriched upon trr knockdown were also enriched in the analysis of G9a mutant heads) (**S2 Fig**). Taken together, this analysis suggests that most of the biological convergence between dysregulated transcriptomes in *trr* and *G9a* mutant conditions happens via indirect regulation. This convergence could result from misregulation of a limited number of common direct targets, or by misregulation of different direct target genes that act in common biological pathways.

In summary, whole-transcriptome analysis revealed a large and significant overlap between genes and biological pathways that are differentially expressed upon loss of trr and G9a, suggesting functional convergence between these two proteins in the fly brain.

## Discussion

Dynamic regulation of gene expression is essential for brain development and function. To date, at least 68 genes encoding chromatin regulating proteins have been implicated in the etiology of ID [38–40]. It is possible that disruption of chromatin related biological networks through mutations in different genes results in an overlapping phenotypic spectrum in the corresponding disorders. Indeed, several chromatin-related disorders, such as Pitt-Hopkins syndrome, Smith-Magenis syndrome, Rett syndrome, *MBD5* deletion/duplication, and Angelman syndrome, have a clear clinical overlap characterized by ID/ASD and additional behavioral and neurological problems [7, 8]. Here, we describe five patients with *de novo* heterozygous loss of function mutations in *KMT2C*, and demonstrate a clinical overlap with this group of ID/ASD disorders (**Fig 1**, **Table 1**). In *Drosophila*, we demonstrate molecular convergence between trr, the KMT2C/D ortholog, and G9a, the ortholog of EHMT1, which is involved in Kleefstra syndrome. The observed clinical and molecular convergence between the KMT2 and EHMT protein families suggests that they are important players in an overlapping gene regulatory network that is critical for normal neuronal development and function.

### KMT2 and EHMT histone methyltransferase families converge in the regulation of adult brain function

An increasing body of evidence suggests that chromatin modifiers contribute to gene regulatory networks that are important for complex brain processes like learning and memory. The molecular activity and protein interaction partners of trr have been well described, but only for early developmental stages and in cultured S2 cells [14, 25, 28]. Using conditional knockdown we showed that trr is required in post-mitotic MB neurons for normal short term courtship memory (**Fig 2L**). We did not observe any gross morphological defects in the MB upon trr knockdown (**Fig 2I and 2J**), and although we cannot rule out a subtle role for trr in MB morphogenesis, this suggests that trr may regulate behaviour and memory through a role in the post-developmental functioning of adult MB neurons. One key interaction partner for trr is the ecdysone receptor (EcR), a nuclear hormone receptor that binds to 20-hydroxyecdysone and requires trr-mediated H3K4me3 for optimal gene activation [14]. 20-hydroxyecdysone is actively synthesized in response to courtship conditioning and is required for long term courtship memory [41], supporting the idea that trr may be involved in the acute regulation of adult brain function through its role in EcR-mediated transcription. Indeed, thirteen known ecdysone responsive genes were identified in our trr genomic binding sites (**S2 Table**), consistent with the known trr-EcR interaction. *Drosophila* G9a is also required in adult neurons for normal courtship memory [18], and the mouse G9a orthologs, Ehmt1 and Ehmt2, have been implicated in postnatal regulation of fear memory [24]. Interestingly, the specific histone modifications that are deposited by the EHMT and KMT2 protein families (H3K9me2 and H3K4me3, respectively), are among the very few forms of histone methylation that are dynamically regulated in the rat brain in response to fear conditioning [42, 43]. Together, the evidence points towards a role for the EHMT and KMT2 families in adult brain plasticity, through active regulation of histone modifications and gene expression.

### Molecular convergence of *Drosophila* G9a and trr

Here, we describe a high level of convergence in genes that are differentially expressed in *trr* and *G9a* mutant fly heads (**Fig 4E**). Although this overlap is significantly more than expected by random chance, the match at the level of biological processes is even more striking. There is a nearly perfect overlap in the enriched GO terms between the two independent mutant conditions (bold GO-terms in **Fig 4B and 4D**). This suggests that trr and G9a may regulate; (1) distinct gene sets that operate in similar biological pathways, and /or (2) a limited number of joint key target genes that influence these pathways. In total, we observed five potential hub genes that are differentially expressed in both mutant conditions and identified in ChIP experiments as potential direct targets for both G9a and trr (**S9 Table**). Two of these genes, *pyruvate carboxylase* (*PCB*), and *mitochondrial transcription termination factor* (*mTTF*), have a clear link to mitochondrial metabolism [44, 45], therefore, their misregulation could affect aspects of metabolism that are reflected in the GO terms associated with the differentially expressed genes in *trr* and *G9a* mutant heads (**Fig 4**). Loss of function mutations in PCB give rise to several disorders that include developmental delay, ID, and seizures, and its misregulation may thus contribute to manifestation of these features in *EHMT1* and *KMT2C* patients.

Interestingly, four of the five potential hub genes (*Reg-2*, *ACER*, *PCB*, and *Arc-1*) have a direct or indirect link to memory formation. Reg-2 and ACER are both linked to circadian rhythm [46, 47], which is known to be important for memory formation (reviewed in [48, 49]). The metabolic protein PCB has been linked to age-induced memory impairment due to the cumulative damaging effect of metabolic free radicals in the aging brain [44]. Arc-1 is the ortholog of mammalian Arc (activity regulated cytoskeletal protein), an immediate early gene that is activated in response to neuronal activity associated with learning and memory [50]. Arc is localized at the postsynaptic density and regulates many forms of synaptic plasticity including LTP, LTD and homeostatic synapse scaling [51]. A recent study has implicated mouse Ehmt1 in repression of Arc transcription in response to homeostatic synaptic scaling [52]. Arc was predicted to be indirectly regulated by Ehmt1 based on a lack of differential dimethylation of H3K9 at the *Arc* promoter [52]. Our data suggests that *Drosophila* G9a deposits H3K9me2 at the 3-prime end of *Arc-1* [18], which has not been tested in mouse. Although, this might reflect a differential mechanism of gene regulation between *Drosophila* and mammals, it appears that *Arc* and *Arc-1* represent an evolutionarily conserved target for EHMT proteins. Having identified *Arc-1* as a trr target gene in *Drosophila*, it will be interesting to see if this evolutionary relationship also holds true for KMT2 proteins.

It is possible that direct misregulation of specific genes, like *Arc-1*, may underlie cognitive defects in human and *Drosophila* with mutations in *KMT2* and *EHMT* genes. However, our analysis of candidate genomic target genes in *Drosophila* revealed hundreds of other genes that may be involved. At the level of genomic targets, we observed more than 1000 genes that are potentially regulated by both G9a and trr. These genes show a strong enrichment for neuronal GO terms, such as “synapse assembly”, “brain development”, and “cognition” (**S4 Table**). Notably, “negative regulation of Ras protein signaling”, the most enriched GO-term (**Fig 3F**), is linked directly to a subset of ID disorders known as RASopathies [37]. However, the relevance of these targets remains in question as we do not observe differential expression for most of these genes in G9a and trr mutant fly heads, at least in the steady state conditions examined here. This, however, does not exclude the possibility that the histone modifications mediated by trr and G9a may poise these genes for transcriptional regulation. It is possible that trr- or G9a-mediated regulation is only relevant in specific cells or in response to certain stimuli, such as the formation or retrieval of memory. Such activity-dependent or cell-specific gene expression mechanisms are likely to escape detection in our analyses in which steady-state expression is determined in whole heads.

### Clinical convergence in chromatin-related ID disorders

The investigation into the molecular convergence between EMHT and KMT2 proteins in this study was motivated by the identification of an overlapping clinical phenotype resulting from mutations in these genes. This convergence likely goes beyond EHMT1/G9a and KMT2C/trr. Apart from KMT2C, mutations in additional components of the well-defined COMPASS protein complex, including the KMT2C paralog KMT2D, and the histone demethylase KDM6A, are causative for Kabuki syndrome, another ID disorder which shares autistic behavior and developmental delay with Kleefstra syndrome and the patients described here [8]. Molecular and clinical convergence has also been demonstrated between Pitt-Hopkins syndrome (causative gene *TCF4*) and Kleefstra syndrome [8, 53]. Further fundamental research will likely reveal additional molecular connections between genes that are mutated in other ID/ASD disorders showing clinical overlap with KMT2C haploinsufficiency. Promising candidates to be involved in a common molecular network include *TCF4* [54], *RAI1* [55], *MECP2* [56], *MBD5* [57, 58], *KMT2D* [59], *UBE3A* [60], which are all ID genes encoding proteins involved in gene regulation. Expanding our knowledge of these molecular networks may help to understand pathways underlying ID/ASD that could be exploited in the development of therapies for genetically distinct but clinically related disorders.

## Materials and methods

### Identification and prioritization of variants

Individuals 1–4 had unexplained ID or developmental delay and were ascertained through family-based whole exome sequencing in a diagnostic setting in the Department of Human Genetics at Radboudumc and MaastrichtUMC. Exome sequencing and data analysis were performed as previously described in the probands and their unaffected parents [29]. Individual 5 was ascertained during a clinical genetic workup due to failure to thrive and lack of expected normal childhood development at Children's Hospital Colorado (Aurora, Colorado). Chromosomal microarray analysis for individual 5 was performed using both copy number and single-nucleotide polymorphism (SNP) probes on a whole genome array (CytoScan HD platform; Affymetrix, Santa Clara, CA). The *KMT2C* deletion was confirmed to be *de novo* by parental microarray testing. Additionally, this deletion was confirmed using locus-specific qPCR. The primer pairs used were directed to *KMT2C* and two adjacent negative control regions (**S10 Table**). All data were analyzed and reported using the February 2009 (hg19) human genome build.

Although all individuals harbored *de novo* loss of function mutations in *KMT2C* (**Fig 1A**), exome results revealed that individual 1 and 2 carry one or two additional *de novo* mutations that do not appear to be related to the core phenotype of the individuals presented here. Individual 1 carried a second *de novo* truncating mutation in *PHF21A*, which is located in the 11p11.2 contiguous gene deletion syndrome named Potocki-Shaffer syndrome, PSS (OMIM #601224) [61, 62]. Three individuals with translocation breakpoints through *PHF21A* were reported previously and it was concluded that this gene was associated with the PSS. However, no intragenic loss of function mutations are reported in association with this syndrome so far. The mutation in individual 1 is present in the last exon of the gene and is therefore not subjected to nonsense mediated RNA decay, however it is possible that this variant may contribute to the phenotype observed in this patient.

In individual 2 we found the *KMT2C* change c.7550C>G (p.Ser2517*) in mosaic in blood. Individual 2 carries two additional *de novo* missense mutations in *C11orf35* (p.Pro602Leu) and *UBR5* (p.Arg1907His). Protein function or mutations associated with other diseases for *C11orf35* are not known, and thus we cannot rule out that this variant may contribute to the patient phenotype. With respect to *UBR5*, the same mutation has been previously described in a family with adult myoclonic epilepsy [63], but that family was not associated with ID/Neuro Developmental Delay. The missense change in *UBR5* individual 2 is therefore unlikely to cause his ID and autism. Moreover, no epilepsy is present in this individual at age 10.

### Fly stocks and genetics

Flies were reared on standard cornmeal-agar media at 25°C on a light/dark cycle of 12h/12h in 50% or 70% humidity. The following stocks were obtained from Bloomington Drosophila Stock Center: *UAS-trr-RNAi* generated by the Transgenic RNAi Project (TRiP) (stock #36916: y1 sc*v1;P{TRiP.*HMS01019}attP2*), *UAS-mCD8*::*GFP* (Stock #5137: y [1] w [*]; P{w [+mC] = UAS-mCD8::*GFP*.*L}LL5*, *P{UAS-mCD8*::*GFP*.*L}2*), *UAS-mCherry-RNAi* (stock #35785: y [1] sc [*] v [1]; P{y [+t7.*7] v [+t1*.*8] = VALIUM20-mCherry}attP2*), *Elav-Gal4; UAS-dicer2* (stock #25750: *P{w [+mW*.*hs] = GawB}elav [C155] w [1118]; P{w [+mC] = UAS-Dcr-2*.*D}2*), and *R14H06-Gal4* (stock #48667: *P{GMR14H06-GAL4}fattP2)* [33]. *G9a*^DD1^ mutants and the precise excision control were generated previously [18]. To study the effects of trr down regulation, the UAS/Gal4 system was used with *UAS-trr-RNAi* combined with pan-neuronal (*elav-Gal4*) or mushroom body (MB) specific (*R14H06-Gal4*) Gal4 driver lines. For all *trr* knockdown experiments, genetic control animals without the RNAi hairpin were generated using the (AttP2) genetic background control strain (Bloomington #36303) or the *mCherry-RNAi* strain (Bloomington #35785). For trr ChIP-seq experiments, a Nijmegen wild type strain was used.

### Imaging of mushroom bodies and immunohistochemistry

*R14H06-Gal4* combined with *UAS-mCD8*::*GFP* was used to visualize the calyx region of the *Drosophila* MB in controls and *trr* knockdown flies. The following genotypes were analyzed: *UAS-mCD8*::*GFP/+;R14H06-Gal4/UAS-trr-RNAi* (trr knockdown) and *UAS-mCD8*::*GFP/+;R14H06-Gal4/+* (control). Adult brains were dissected in PBS (pH 7.2) and fixed with ice cold methanol for 2 minutes, washed three times with PBS for 5 minutes each and permeabilized in PBS with 0.3% Triton-X 100 (PBT) for 1 hour. Fixed and permeabilized brains were blocked (5% normal goat serum (NGS)) at room temperature for 2 hours and incubated in 300nM DAPI solution for three minutes, followed by three five minute washes in PBT. Next, brains were incubated with the primary antibody (rabbit anti-trr [24], 1:5000) in PBT with 5% NGS for 72 hour at 4°C followed by five 20 minute washes in PBT. Brains were incubated with the secondary antibody (goat anti-rabbit Alexa Fluor 568, 1:250; Invitrogen) for 48 hour in PBT with 5% NGS at 4°C and washed at room temperature in PBT five times for 20 minutes. Brains were mounted in Vectashield (Vector Laboratories) and imaged on a confocal microscope (Zeiss LSM 510 duo vario confocal microscope). Confocal stacks were processed using ImageJ software [64].

### Courtship conditioning assay

Courtship conditioning assays were performed on 5-day-old males raised at 25°C, 70% humidity, and a 12h day/night rhythm as previously described [18, 35]. The following genotypes were analyzed: *R14H06-Gal4/UAS-mCherry-RNAi* (control) and *R14H06-Gal4/UAS-trr-RNAi* (trr-RNAi). These genotypes were generated by crossing females containing the trr-RNAi or mCherry-RNAi inserted into the TRiP attP2 genetic background (genotypes: trr RNAi—y[1] sc[*] v[1]; P{y[+t7.*7] v[+t1*.*8] = TRiP*.*HMS01019}*, mCherry RNAi—y[1] sc[*] v[1]; P{y[+t7.*7] v[+t1*.*8] = VALIUM20-mCherry}attP2)*, with identical males containing the Gal4 driver (genotype: *P{GMR14H06-GAL4}fattP2*. Males were randomly assigned to either trained (n_*control*_ = 59, n_*trr-RNAi*_ = 62) or naïve (n_*control*_ = 56, *n*_*trr-RNAi*_ = 60) groups. Training was performed by pairing individual males in with 5-day-old mated wild type females. All experiments were conducted with a one-hour training period, and tested after a one-hour isolation period. For each fly pair, a courtship index (CI) was calculated as the percentage of a 10 minute time period spent courting. Courtship behaviour was manually quantified by observing videos for all 237 fly pairs analyzed in this study. Quantification was performed by trained observers that were naïve to the nature of the experiment. Comparisons of average CI between naïve and trained groups of the same genotype were calculated using a Mann-Whitney test. A learning index (LI = (CI_naive-_CI_trained_)/CI_naive_) was calculated to compare courtship memory between genotypes. Statistical comparisons between genotypes were conducted using a randomization test [65] with a custom bootstrapping script created in R (10,000 replicates) [66].

### ChIP-seq and identification of trr binding sites

Chip was performed using an antibody directed against trr [14, 34]. This antibody was originally validated by the lack of staining in trr homozygous null mutant embryos [14]. Additionally, this antibody shows complete lack of signal in mutant clones of the *Drosophila* salivary gland, and has been used for ChIP experiments showing very specific trr binding patterns at EcR response genes during ecdysone-mediated developmental transitions [34]. Here, we provide additional evidence for specificitiy of this antibody by showing reduced immunofluorescent staining in mushroom body target cells expressing a trr-RNAi construct (**Fig 2**).

Chromatin was extracted from 50 μL aliquots of frozen wildtype fly heads, aged between 0 and 5 days old, in biological duplicates. Fly heads were crushed in PBS (Sigma) and crosslinked with formaldehyde (Sigma) at a final concentration of 1% for 30 minutes. Crosslinking was terminated using glycine (Invitrogen) at a final concentration of 125mM and crosslinked material was immediately washed twice in PBS and centrifuged at 13000rpm, for 15 minutes at 4 degrees. The pellet was resuspended in buffer 1 (15 mM Tris-HCl (pH 7.5), 60 mM KCl, 15 mM NaCl, 1 mM EDTA, 0.1 mM EGTA, 0.15 mM spermine, 0.5 mM spermidine, 0.1 mM sucrose) and the solution was homogenized using a QiaShredder column (Qiagen). Cells were lysed using buffer 1 supplemented by 2% triton-X-100 (Sigma) and a crude nuclear extract was collected by centrifugation (6000 rpm, 10 minutes at 4 degrees). Nuclei were re-suspended in incubation buffer (0.15% SDS, 1% triton x-100, 150 mM NaCl, 1 mM EDTA, 0.5 mM EGTA, 10 mM Tris) supplemented with 0.1% BSA (Sigma) and 1x protease inhibitor (Roche) and subjected to sonication (Diagenode Bioruptor) for 30 minutes (30 seconds on/off cycle using the “high intensity” mode), yielding average DNA fragments of 150–300 base pairs. Immunoprecipitation reactions were performed overnight in incubation buffer with 3 ng of anti-trr antibody (gift from Dr. A. Mazo [14]), protease inhibitor cocktail (Roche), BSA, and pre-blocked protein A/G agarose beads (Santa Cruz). Chromatin-antibody-bead complexes were recovered by centrifugation (4000 rpm, 2 minutes at four degrees) and washed twice with low salt buffer (0.1% SDS, 1% Triton, 2 mM EDTA, 20 mM Tris pH 8, 150 mM NaCl), once with high salt buffer (0.1% SDS, 1% Triton, 2 mM EDTA, 20 mM Tris pH 8, 500 mM NaCl), once with LiCl wash buffer (10 mM Tris pH 8.0, 1% Na-deoxycholate, 1% NP-40, 250 mM LiCl, 1 mM EDTA) and twice with TE buffer. Chromatin was eluted in 1% SDS, 0.1M NaHCO_3_, 200 mM NaCl and de-crosslinked at 65° Celsius (C) for four hours. DNA was purified by phenol/chloroform extraction and ethanol precipitated using linear acrylamide (Ambion) and sodium acetate at -20 degrees. Library preparation for Illumina sequencing was performed using the Truseq DNA sample prep kit V2 (Illumina) with approximately 3 ng of starting DNA 15 PCR cycles for amplification. Library fragment size was assessed using the 2100 Agilent Bioanalyser and was shown to be between 200 and 400 bp. Cluster generation and sequencing-by-synthesis (50bp) was performed using the Illumina Hiseq 2000 according to standard protocols of the manufacturer. The image files generated by the HiSeq were processed to extract DNA sequence data. We obtained between 30 and 54 million reads per sample. Reads were mapped to the *Drosophila* genome (BDGP R5/dm3) using the Burrows Wheeler Aligner (BWA, version 0.6.1) with standard settings allowing 1 mismatch [67]. Total alignment efficiency was more than 95%. Duplicate reads and reads with a mapping quality score (MAPQ) below 15 were excluded from downstream analysis (**S1 Table**). For each biological replicate, trr binding sites were identified using MACS2 [36] with input DNA as control (**S1 Table**). Bindings sites on chromosome U, Uextra and mitochondrial genome were not used for further analysis. Remaining putative trr binding regions were visualized in a heatmap that was sorted based on k-means clustering of read intensity around the centre of the peak using the python script fluff_heatmap.py (https://github.com/simonvh/fluff) (**S3A Fig**). Individual clusters were examined visually in the genome browser to assess the quality of the peaks within the clusters. Two clusters (8 and 15) of replicate 1 were identified that contained many false positive peaks with a relatively small binding region and cluster 15 had very few reads around the centre. Peaks within these clusters were removed from downstream analysis. For trr_rep2 all clusters appeared to represent high quality trr binding sites. Trr binding regions from both biological replicates were merged and concatenated and the number of reads present in these regions were counted in each sample individually using HTSeq [68] and normalized to library size. The ratio and mean of reads in each binding region was calculated between the two biological duplicates to identify trr binding sites that were consistent between the two biological replicates. We included all binding sites with a mean number of reads > 100 and < 2 fold difference in normalized read count between the two biological replicates (**S3B Fig**). Using these criteria we identified 3371 predicted trr binding regions, which appeared to be very consistent upon visual inspection in the UCSC genome browser (**S3D Fig**). These predicted trr binding sites were allocated to the nearest genomic feature using the pearl script annotatePeaks.pl [69] with standard settings. In order to find out if detected binding sites of trr are overrepresented in any genomic regions, we compared trr binding sites to an equal set of randomly selected genomic positions using https://www.random.org/ (**Fig 3B**). The average trr occupancy profile over all transcription start sites (**Fig 3C**) was calculated using ensemble annotations from release 84 [70] with read count data generated by HTseq-count [68] in 50-bp bins spanning all transcription start sites.

To validate the ChIP-seq data, independent ChIP reactions were performed as described above and tested by qPCR. Validation targets were selected based on ChIP-seq data with representative binding regions on the promoters of *mor*, *lis-1*, *Atg9*, *Hsc70-4*, *Socs36e*, *Tsp42ed*, *smid* and *med21*. Additionally, we selected two trr-negative regions in the promoters of *drm99B* and *CG1646* (**S10 Table**). All primers were tested and approved for amplification efficiency according to standard methods. qPCR was performed on the ChIPed and input samples using SYBRgreen master mix (Promega) and the 7900HT Fast Real Time PCR system (Applied biosystems) according to the manufacturer’s instructions. Fold enrichments per target were calculated using the mean of the percent input of the two negative regions relative to the positive region. Mean fold enrichments are plotted with standard error of the mean as error bars (**S3C Fig**). All regions tested confirmed the ChIP-seq results. In addition we compared our trr ChIP-seq targets to published trr ChIP-seq data from cultured *Drosophila* S2 cells that was obtained using a different trr antibody [25]. In S2 cells, 1482 genes were identified with a trr peak at the promoter [25]. 943 of these genes were also identified as trr targets in this study. This is a very high overlap considering the vastly different starting material (cultured S2 cells versus fly heads), suggesting a high degree of concordance between the two antibodies.

### Transcriptional profiling

Mutant and control lines for *trr* and *G9a* were aged between 1 and 5 days and snap frozen in liquid nitrogen. Frozen fly heads were harvested by vortexing and separated from other body parts through a series of standard laboratory sieves. Total RNA was extracted from 50 μL aliquots of frozen fly heads using the RNAeasy lipid tissue mini kit (Qiagen). For *trr* knockdown, two biological replicates were used, for *G9a* mutants, three biological replicates were used for each condition. mRNA was purified using the Oligotex kit (Qiagen) and cDNA was synthesized using the SuperScript III First Strand synthesis kit (Thermo Fisher) using random hexamers as primers. Second strand cDNA was synthesized using *E*. *Coli* polymerase and T4 ligase (New England Biolabs Inc. (NEB)). Remaining RNA was removed using 2 units RNaseH (NEB) before the cDNA was purified using the MinElute PCR purification kit (Qiagen). DNA end repair was performed followed by ligation of Illumina sequencing adaptors and size selection for 300 bp by 2% E-gel (Invitrogen). Fragments were linearly amplified (15 PCR cycles), as validated by quantitative real-time PCR (qPCR), and sample quality was assessed using the Agilent Bioanalyser 2100. Cluster generation and sequencing-by-synthesis (36bp) was performed using the Illumina Genome Analyser IIx (GAIIx) according to standard protocols of the manufacturer. The image files generated by the GAIIx were processed to extract DNA sequence data. From the GAIIx, we obtained between 27 and 35 million reads. Reads were mapped to the *Drosophila* genome (BDGP R5/dm3) using the Burrows Wheeler Aligner (BWA, version 0.6.1) with standard settings allowing 1 mismatch [67]. Only the uniquely mapped reads were used for further analysis and total alignment efficiency was between 63% and 79% for G9a samples and between 79% and 81% for trr samples (**S5 Table**). Total read count data was generated by the python script HTSeq-count (http://www-huber.embl.de/HTSeq/doc/overview.html) with gene annotations extracted from the file Drosophila_melanogaster.BDGP5.75.gtf, available at http://www.ensembl.org. In all samples, over 96% of aligned reads, mapped unambiguously to exons. The unambiguously mapped reads, ranging from 18 to 25 million reads for G9a samples and from 26 to 28 million reads for trr samples, were used for further analysis of differential gene expression by DeSeq2 [71]. Hierarchical clustering based on Euclidean distances with Pearson correlation using the normalized expression values and variance stabilizing transformation, revealing a high degree of similarity between biological replicates (**S4A and S4C Fig**). In order to perform statistical comparisons, dispersion values were estimated using the DESEQ method. As expected, we observed a high degree of correlation between gene expression and dispersion values with decreasing dispersion upon increasing expression patterns (**S4B and S4D Fig**). We then used DESeq2 to identify genes that are differentially expressed in mutant fly heads based on negative binominal distribution (adjusted p-value < 0.05, fold change > 1.5, **Fig 4A and 4C, S6 and S7 Table**).

Validation of *trr* knockdown was performed using SYBRgreen master mix (Promega) and the 7900HT Fast Real Time PCR system (Applied Biosystems) according to the manufacturer’s instructions on a new biological replicate cDNA (described above) performed in triplicate technical replicates. Beta-cop and RP49 were used as reference genes for normalization and calculation of fold change differences upon pan-neuronal knockdown in fly heads using the ΔΔ Ct procedure (**S1 Fig**). All RT-qPCR primers (**S10 Table**) are validated for amplification efficiency according to standard procedures.

### Gene ontology enrichment and hypergeometric analysis

Gene Ontology (GO) enrichment analysis was performed using the Panther software version 11.1 [52] on http://geneontology.org/ (GO Ontology database released on 2015-08-06 and 2016-10-24) using the GO-SLIM function for GO enrichment datasets that showed a high degree of redundancy (**Fig 4 and S2 Fig**). For GO enrichment analysis of differential gene expression, list was used with genes expressed in fly heads as background control. This list was generated by exclusion of 4579 genes that had less than 10 reads, leaving 11103 genes. Overlap between datasets was determined and visualized as a Venn diagram by BioVenn [72] (**Figs 3E and 4E**). Hypergeometric statistics on overlaps were calculated using https://www.geneprof.org/GeneProf/tools/hypergeometric.jsp.

### Accession numbers

The raw data for RNA-seq and ChIP-seq is available at the NCBI Gene Expression omnibus (GEO), accession number GSE89459.

### Ethics statement

Diagnostic whole exome sequencing (WES) was approved by the medical ethics committee of the Radboud University Medical Center, Nijmegen, The Netherlands (registration number 2011–188). For all patients, written informed consent for WES was obtained after counseling by a clinical geneticist.

## Supporting information

S1 Fig Pan-neuronal knockdown of *trr* in fly heads. Quantification of relative trr expression by qPCR in heads of control and *elav-Gal4* knockdown flies. Error bars represents SEM.
(TIF)

S2 Fig GO enrichment analysis of “potential direct” and “potential indirect” trr and G9a target genes. (A-D) All enriched gene ontology (GO) terms identified using the Panther software (GO-slim setting) for potential direct trr (A) and G9a (B) target genes, and potential indirect and trr (C) and G9a (D) targets. Panther overrepresentation test from GO database version 11.1 (released 2016-10-24).
(TIF)

S3 Fig Filter criteria and validation of trr ChIP-seq. (A) Flow chart describing the filtering steps for identification of trr binding sites. After ChIP-seq trr binding regions were identified using MACS2 and clustered using the python script fluff_heatmap.py. Clusters with low quality peaks were removed and the remaining peaks were merged and concatenated. The number of reads in each trr binging regions was quantified with HTSEQ-count. (B) Scatter plot of the average number of reads in each trr binding region against the ratio of reads between the two biological replicates. Black dots represents the 3371 high confident peaks with mean number of reads > 100 and ratio < 2, suggesting consistency between the two biological replicates. (C) Bar graph showing ChIP-qPCR validation of trr binding regions identified by ChIP-seq. Fold enrichment is calculated over negative control regions, relative to the input. Error bars represent SEM of three biological replicates. (D) Screenshot of tracks from the UCSC genome browser showing two trr ChIP-seq replicates and the input control.
(TIF)

S4 Fig Quality control for RNAseq and differential expression analysis. (A,C) Dendrogram and heat map illustrating euclidean distances and Pearson correlation between genome wide mRNA expression levels in G9a mutants (A) and trr knockdown (C) heads, compared to the respective controls. (B,D) Scatter plots showing dispersion estimates as determined using DESeq2, plotted against the mean of normalized reads for EMHT (B) and trr (D) RNA-seq datasets (black dots—gene-wise maximum-likelihood estimates, red dots—fitted values, blue dots—final dispersion. Genes with dispersion outliers were not used for further analysis. Note the decreasing dispersion values as the gene expression increases.
(TIF)

S1 Table ChIP-seq depth, alignment, mapping efficiency and MACS2 settings. Total number of reads, and the percentage aligned reads are shown. Next, the percentage of aligned reads to unambiguous places relative to total aligned reads and the percentage of reads with MAPQ scores higher than 15 are shown. Lastly, the total number of high quality reads that was used for analysis is shown.
(XLSX)

S2 Table Annotation of trr ChIP-seq peaks to nearest genomic feature using HOMER software (Raw data to Fig 3A). (XLSX)

S3 Table Gene ontology analysis of trr promoter associated genes (Raw data to Fig 3D). (XLSX)

S4 Table Gene ontology analysis of overlap between trr binding sites and predicted G9a target genes (Raw data to Fig 3F). (XLSX)

S5 Table RNA-seq depth. Alignment and mapping efficiency of trr- and G9a mutant samples. Shown are the total number of reads, and the percentage aligned. Next, the percentage of aligned reads relative to the total number of aligned reads and unambiguous mapped reads are shown. Lastly, the total number of high quality reads that was used for analysis is shown.
(XLSX)

S6 Table Differential expressed genes in trr mutant (Raw data to Fig 4A). (XLSX)

S7 Table Differential expressed genes in G9a mutant (Raw data to Fig 4C). (XLSX)

S8 Table Statistical analysis of up and down regulated genes that are differentially expressed genes in both trr and G9a mutant fly heads. (XLSX)

S9 Table Gene ontology annotations for the five potential direct targets of both G9a and trr. (XLSX)

S10 Table List of primers used in this study. (XLSX)
