# Supplementary figures and images for: Functional convergence of histone methyltransferases EHMT1 and KMT2C involved in intellectual disability and autism spectrum disorder
Source: PLoS Genet. 2017 Oct 25;13(10):e1006864. doi: 10.1371/journal.pgen.1006864 (PMC5656305; doi:10.1371/journal.pgen.1006864)

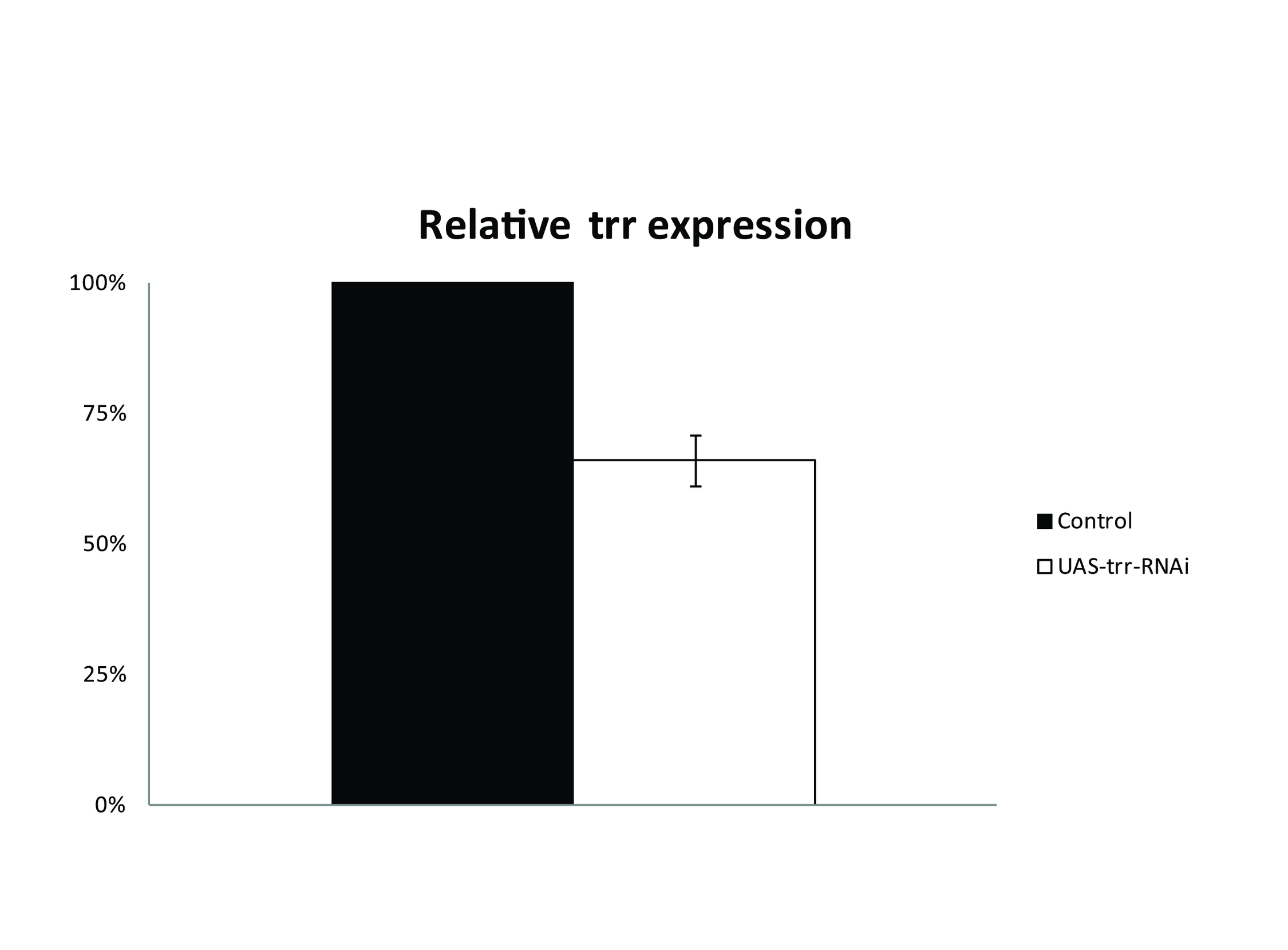

Supplement: S1 Fig — Quantification of relative trr expression by qPCR in heads of control and elav-Gal4 knockdown flies. Error bars represents SEM. (TIF) [file pgen.1006864.s001.tif]

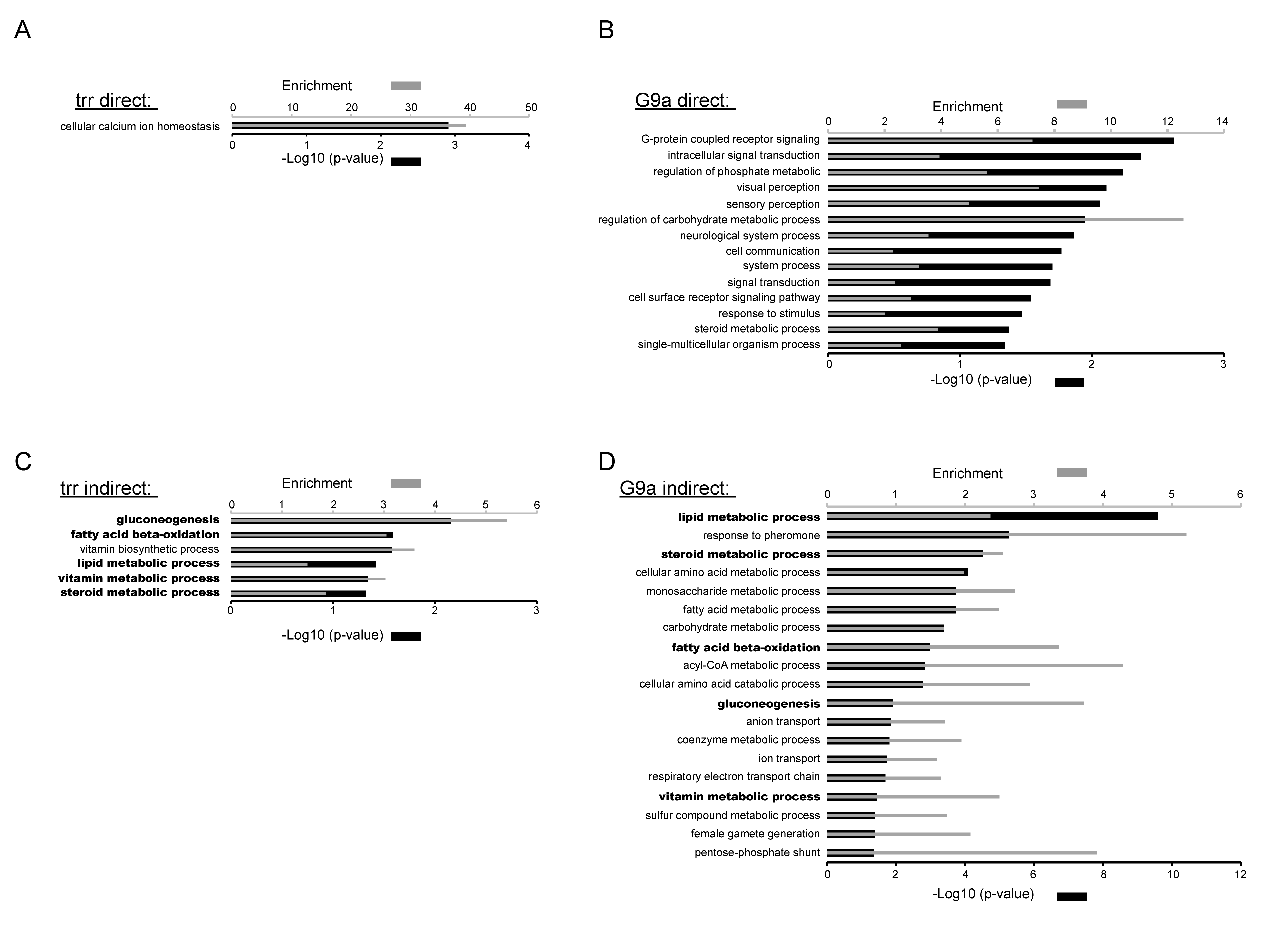

Supplement: S2 Fig — (A-D) All enriched gene ontology (GO) terms identified using the Panther software (GO-slim setting) for potential direct trr (A) and G9a (B) target genes, and potential indirect and trr (C) and G9a (D) targets. Panther overrepresentation test from GO database version 11.1 (released 2016-10-24). (TIF) [file pgen.1006864.s002.tif]

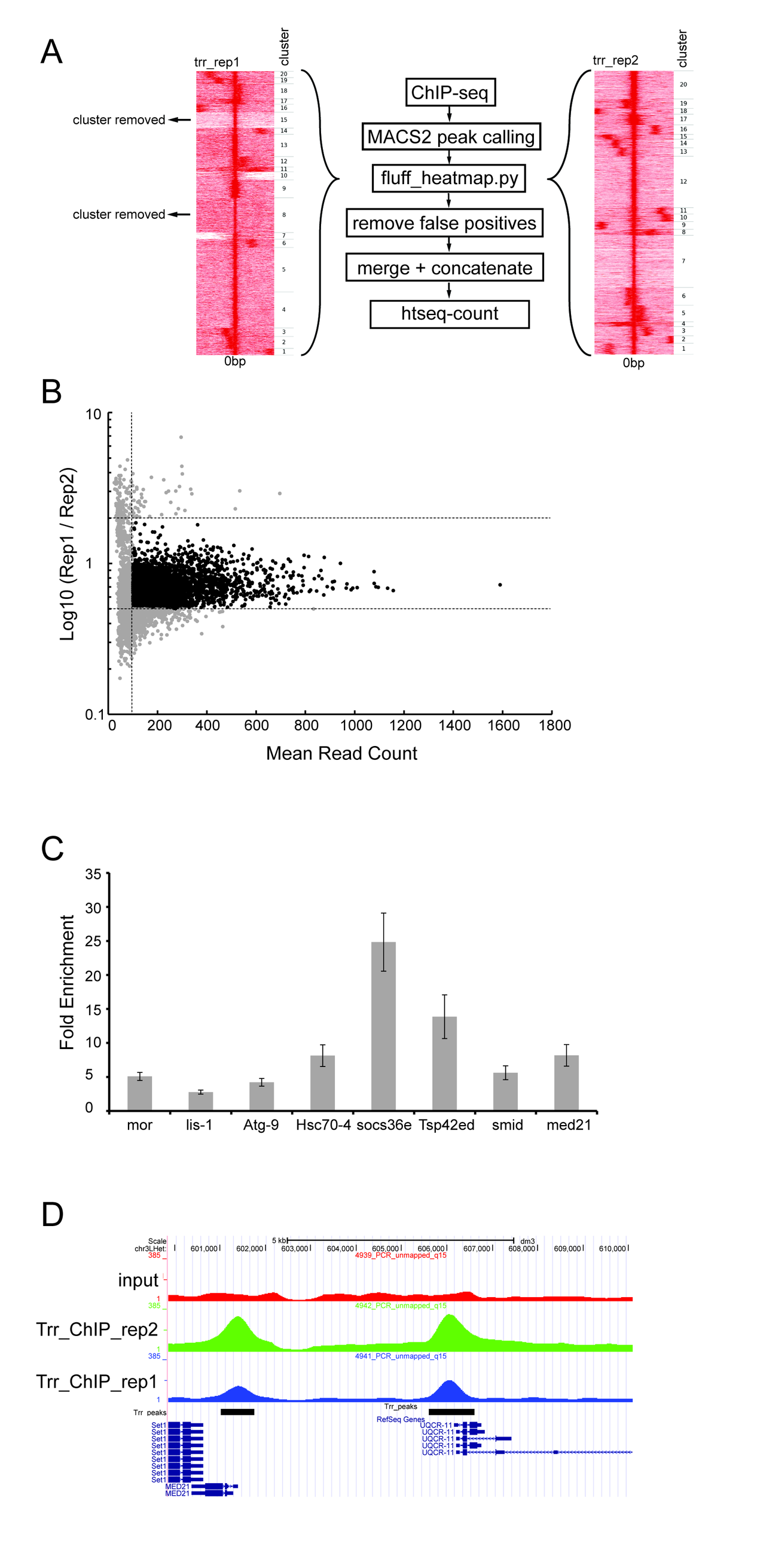

Supplement: S3 Fig — (A) Flow chart describing the filtering steps for identification of trr binding sites. After ChIP-seq trr binding regions were identified using MACS2 and clustered using the python script fluff_heatmap.py. Clusters with low quality peaks were removed and the remaining peaks were merged and concatenated. The number of reads in each trr binging regions was quantified with HTSEQ-count. (B) Scatter plot of the average number of reads in each trr binding region against the ratio of reads between the two biological replicates. Black dots represents the 3371 high confident peaks with mean number of reads > 100 and ratio < 2, suggesting consistency between the two biological replicates. (C) Bar graph showing ChIP-qPCR validation of trr binding regions identified by ChIP-seq. Fold enrichment is calculated over negative control regions, relative to the input. Error bars represent SEM of three biological replicates. (D) Screenshot of tracks from the UCSC genome browser showing two trr ChIP-seq replicates and the input control. (TIF) [file pgen.1006864.s003.tif]

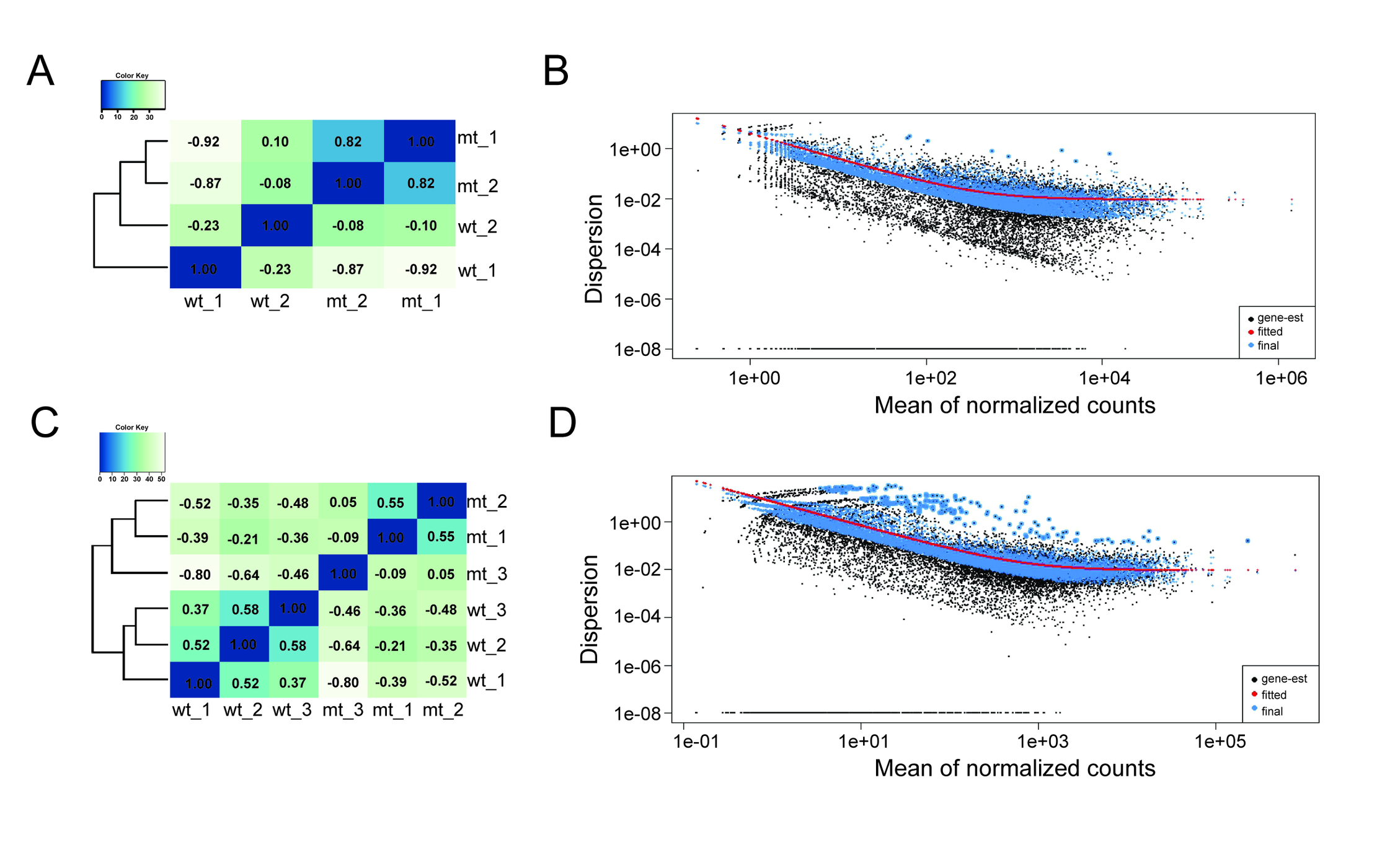

Supplement: S4 Fig — (A,C) Dendrogram and heat map illustrating euclidean distances and Pearson correlation between genome wide mRNA expression levels in G9a mutants (A) and trr knockdown (C) heads, compared to the respective controls. (B,D) Scatter plots showing dispersion estimates as determined using DESeq2, plotted against the mean of normalized reads for EMHT (B) and trr (D) RNA-seq datasets (black dots—gene-wise maximum-likelihood estimates, red dots—fitted values, blue dots—final dispersion. Genes with dispersion outliers were not used for further analysis. Note the decreasing dispersion values as the gene expression increases. (TIF) [file pgen.1006864.s004.tif]
